# Regulation of Tumor Apoptosis of *Poriae cutis*-Derived Lanostane Triterpenes by AKT/PI3K and MAPK Signaling Pathways *In Vitro*

**DOI:** 10.3390/nu15204360

**Published:** 2023-10-13

**Authors:** Shuai Yue, Xi Feng, Yousheng Cai, Salam A. Ibrahim, Ying Liu, Wen Huang

**Affiliations:** 1 College of Food Science and Technology, Huazhong Agricultural University, Wuhan 430070, China; yueshuaihzau@163.com; 2 Department of Nutrition, Food Science and Packaging, San Jose State University, San Jose, CA 95192, USA; xi.feng@sjsu.edu; 3 School of Pharmaceutical Sciences, Wuhan University, Wuhan 430071, China; cysh2002@whu.edu.cn; 4 Department of Family and Consumer Sciences, North Carolina A&T State University, 171 Carver Hall, Greensboro, NC 27411, USA; ibrah001@ncau.edu

**Keywords:** tetracyclic triterpenes, cancer, molecular docking, apoptosis effect

## Abstract

*Poria cocos* is traditionally used as both food and medicine. Triterpenoids in *Poria cocos* have a wide range of pharmacological activities, such as diuretic, sedative and tonic properties. In this study, the anti-tumor activities of poricoic acid A (PAA) and poricoic acid B (PAB), purified by high-speed counter-current chromatography, as well as their mechanisms and signaling pathways, were investigated using a HepG2 cell model. After treatment with PAA and PAB on HepG2 cells, the apoptosis was obviously increased (*p* < 0.05), and the cell cycle arrested in the G2/M phase. Studies showed that PAA and PAB can also inhibit the occurrence and development of tumor cells by stimulating the generation of ROS in tumor cells and inhibiting tumor migration and invasion. Combined Polymerase Chain Reaction and computer simulation of molecular docking were employed to explore the mechanism of tumor proliferation inhibition by PAA and PAB. By interfering with phosphatidylinositol-3-kinase/protein kinase B, Mitogen-activated protein kinases and p53 signaling pathways; and further affecting the expression of downstream caspases; matrix metalloproteinase family, cyclin-dependent kinase -cyclin, Intercellular adhesion molecules-1, Vascular Cell Adhesion Molecule-1 and Cyclooxygenase -2, may be responsible for their anti-tumor activity. Overall, the results suggested that PAA and PAB induced apoptosis, halted the cell cycle, and inhibited tumor migration and invasion through multi-pathway interactions, which may serve as a potential therapeutic agent against cancer.

## 1. Introduction

*Poria cocos* (Schw.) Wolf, as a traditional medicinal and edible resource in China, contains a variety of tetracyclic triterpenes. Pharmacological and clinical studies have shown that *Poria cocos* triterpenes can be used for diuretic, anti-tumor, antioxidant and other purposes [1]. More than 160 terpenoids were obtained from *Poria cocos* by high-speed counter-current chromatography, silica gel column chromatography and preparative high performance liquid chromatography for separation and purification [2]. Currently, more than 70 triterpenoids have been isolated from *Poriae cutis* by Chinese and foreign scholars, and they mainly exist as woolly steroidal tetracyclic triterpenes [3,4].

**Figure 1:**
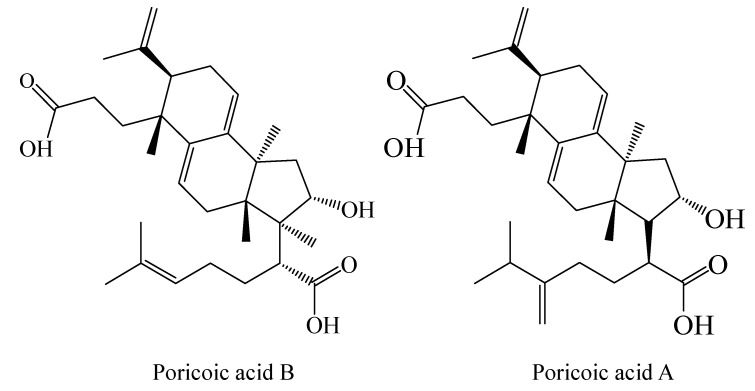
Structures of PAA and PAB isolated from *Poriae cutis*.

Most lanostane triterpenes have been reported to have certain inhibitory activity against tumor cells *in vitro* [5]. The inhibitory effects of lanostane triterpenes on tumor cells are related to their structure [6]. However, due to lanostane triterpene isomers in *Poria cocos*, it is difficult to obtain a large amount of high-purity triterpenoid monomers from *Poria cocos* [7]. In the previous study, four kinds of lanostane triterpene monomers were prepared from *Poriae cutis* by high-speed countercurrent chromatography. Among them, the purity of poricoic acid A and poricoic acid B were both higher than 90%, and their structures are shown in Figure 1.

*Poria cocos* has a strong diuretic effect and is used in the treatment of edema and nephropathy [8]. One of the main components, poricoic acid A, plays an important role in several kidney-related diseases [9]. PAA appears to modulate multiple important signaling pathways, including NF-kB, PI3K/Akt (phosphatidylinositol-3-kinase/protein kinase B), and TGF-b, exerting anti-fibrotic effects *in vivo* and *in vitro* [10,11]. In addition, our laboratory study showed that PAB had higher anti-inflammatory activity than PAA by inhibiting NO production in RAW 264.7 cells induced by lipopolysaccharide (LPS) [12]. Studies have also found that PAA has an inhibitory effect on colon, kidney, prostate and breast cancer cells [13]. It has been shown that PAA induces apoptosis and autophagy in ovarian cancer cells by regulating the mTOR/p70s6k signaling axis [10]. However, the structure–activity relationship of lanostane triterpenoids to inhibiting tumor cells is still unknown, and the mechanisms and pathways of their intervention with tumor cells have not been fully understood. In recent years, molecular docking technology has been widely used in the design of compounds or drugs, drug screening and drug mechanism investigation [14]. In the docking process, the protein and ligand recognize each other, optimize the conformation of the protein and ligand, minimize the free energy of the system, and then predict the affinity and binding pattern between the protein and ligand [15]. In many cases, traditional Chinese medicine treats diseases through multiple compounds and targets, and probing their mechanisms and pathways of action is usually time-consuming and labor-intensive. Therefore, the relevant targets of action of PAA and PAB were initially explored by molecular docking combined with PCR.

In this study, we hypothesized that PAA and PAB can express anti-tumor activities through the interaction of multiple mechanisms. The effects of PAA and PAB on apoptosis, cycle regulation, migration and invasion of tumor cells were investigated. Furthermore, the mechanisms by which PAA and PAB implement their anti-tumor effects was discussed. The possible signaling pathways of the anti-tumor activity of PAA and PAB were discussed and their structure–activity relationships were analyzed and compared.

## 2. Materials and Methods

### 2.1. Extraction and Isolation of Triterpenes

The triterpenes were prepared according to a previously described method [16]. The power of *Poriae cutis* was extracted with 90% (*v*/*v*) ethanol, at a ratio of 1:20, twice at 70 °C, for 2 h. The filtrate was evaporated naturally to dryness, and then dried to obtain the total triterpenes of *Poriae cutis*. Ethyl acetate was used to extract the complete triterpenes three times. By using rotational evaporation at 40 °C, the mixed ethyl acetate extracts were concentrated. The concentrated solution was kept in a freezer at 4 °C without exposure to light for subsequent usage [16]. Then, PAA and PAB were purified by high-speed counter-current chromatography, utilizing a two-phase solvent system of n-hexane, ethyl acetate, methanol, and water (3:6:4:2, *v*/*v*), with the main engine in positive rotation at 800 r/min and a separation flow rate of 3 mL/min. The purity of poricoic acid A and poricoic acid B were 92% and 90%, respectively.

### 2.2. Cell Culture

All cells were bought from the Cell Bank at the Chinese Academy of Sciences (Shanghai, China). Human hepatocellular carcinoma (HepG2) cells were preserved in MEM medium (GIBCO); human lung carcinoma (A549) cells were maintained in DMEM/F12 medium (GIBCO); human colon carcinoma (HT29) cells were cultured in McCoy’s 5A medium (GIBCO); human prostate cancer (DU145) cells were grown in RPMI-1640 medium (GIBCO); human osteosarcoma (SW1353) cells were cultured in DMEM medium (GIBCO). 10% Fetal Bovine Serum (GIBCO) and 1% Penicillin-Streptomycin (GIBCO) were added to the media for all cell lines. All cells were kept in a 5% CO_2_ humidified environment at 37 °C, with two days of fresh medium being added [17]. PAA and PAB were, firstly, configured as 100 mg/mL mother liquor with DMSO and then diluted to 100 μg/mL with a basal medium for subsequent experiments. The cellular experiments were all set up as three independent replica experiments, with three and more parallel wells in a single run.

### 2.3. Antiproliferative MTT Assay

The effects of triterpene compounds on cancer cell proliferative activity were determined using an MTT assay. All cells were cultured in 96-well plates, with an initial density of 2 × 10^4^ cells/well, 24 h before the treatment. After removal of the medium, 100 μL of basal medium containing different concentrations of triterpene compounds or 5 μg/mL of positive control (cisplatin; Sigma, Ronkonkoma, NY, USA) was added and incubated for 24, 48, or 72 h at 37 °C. Each of the treated or control group was contained in six parallel wells. MTT was prepared as a 5 mg/mL stock solution using sterilized PBS and stored at −20 °C. It was diluted tenfold with phenol red-free basal medium for each use. The wells were filled with MTT (0.5 mg/mL), and the plates were then incubated at 37 °C for 4 h. The MTT test was replaced by adding DMSO (150 μL per well) and mixing for 10 min, before measuring absorbance at 490 nm in a multi-well plate reader (Thermo Fisher Scientific, Waltham, MA, USA) [18].

### 2.4. Acridine Orange/Ethidium Bromide (AO/EB) Staining

The HepG2 cells were seeded in Petri dishes (35 mm diameter, Sorfa, Huzhou, China) at 2 × 10^5^ cells/well and treated with triterpene compounds at various concentrations (25 μg/mL, 50 μg/mL and 100 μg/mL) for 48 h. Cells were washed in PBS, stained with AO/EB solution (Leagene Biotechnology Company, Beijing, China) for 15 min at room temperature, and then washed once more in PBS. The optimum excitation/emission parameters for the AO and EB dyes were 488/515 nm and 518/605 nm, respectively. The stained cells were imaged under laser scanning confocal microscopy at 488/561 nm (FV3000, OLYMPUS, Tokyo, Japan).

### 2.5. Apoptosis and Cell Cycle Assay

The HepG2 cells were plated in 6-well plates with a final volume of 2 mL of MEM media with 10% FBS (*v*/*v)* at a density of 2 × 10^6^ cells/mL. Cells were collected and twice washed in PBS after being exposed to triterpene compounds for 48 h. Cells were then resuspended in 1 Binding Buffer (0.1 M Hepes/NaOH (pH 7.4), 1.4 M NaCl, and 25 mM CaCl_2_) at a concentration of 1 × 10^6^ cells/mL. The cells were stained with 10 l of propidium iodide (PI) and 5 μL of Annexin V-FITC, after which 100 μL of the solution was transferred to 1.5 mL culture tubes. The cells were then left to sit at room temperature in the dark for 5 min [18]. Annexin V-FITC was detected by the FITC detection channel (Ex = 488 nm; Em = 530 nm) and PI was detected by the PI detection channel (Ex = 535 nm; Em = 615 nm) using a flow cytometer (Cytoflex-LX, Beckman, Brea, CA, USA). Cells were distinguished by the presence or absence of fluorescence intensity and the strength of the fluorescence signal, and their apoptosis rate was detected.

To detect cell cycle distribution, the HepG2 cell suspension (1 × 10^6^ cells/mL) was mixed with PI in a cultivation tube and then analyzed by flow cytometry [18]. The red fluorescence at the excitation wavelength of 488 nm was recorded. The experimental data were processed by CytExpert Software 2.4.0.

### 2.6. In Vitro Cell Migration/Invasion Assay

The cell migration test was performed on the 24-well transwell plate (Corning Life Science, Tewksbury, MA, USA). After 48 h treatment in culture medium with or without triterpene compounds, HepG2 cells were collected by trypsin digestion, then put back together in serum-free media to bring the cell density to 3 × 10^5^ cells/mL.

The cells were grown in 200 μL of MEM medium in the apical compartment and only 500 μL of medium in the basal compartment, and incubated in 37 °C for 24 h. After incubating, the side of the chamber was gently cleaned with PBS. The cells inside the bottom membrane of the chamber were removed with alcohol cotton swabs. The upper compartment was carefully removed, and the cells were fixed with pre-cooled 2.5% glutaraldehyde for 15–20 min. After 10 min of crystal violet staining, cells attached to the submembrane of the apical compartment were observed under an inverted fluorescence microscope (Ti-S, Nikon, Tokyo, Japan) in five random fields of view [19].

In the cell invasion ability test, the transwell chamber was coated with Matrigel in advance. Matrigel was thawed overnight at 2–8 °C and diluted with a serum-free medium to 2.5 mg/mL. The diluted Matrigel matrix was added to the chamber using a pipette pre-cooled on ice, and collagen I (0.5 mg/mL) was applied to the outside of the bottom membrane of the chamber [19]. The remaining steps were described above.

### 2.7. Intracellular ROS Generation

The HepG2 cells were seeded in all-black 96-well plates and 6-well plates, and treated with or without triterpene compounds in different concentrations for 48 h. The cells were treated with 10 mg/mL DCFH-DA at 37 °C for 30 min after the media were withdrawn [17]. After staining, the cells were digested with trypsin and resuspended in PBS for detection. Cellular fluorescence was evaluated using an inverted fluorescent microscope (Ti-S, Nikon, Japan) and Multiskan Spectrum (HTX, BioTek, Shoreline, WA, USA).

### 2.8. Real-Time Quantitative PCR

Total RNA was extracted using Trizol reagent from HepG2 cells treated with or without triterpene compounds for 24 h (Transgen Biological Technology Co., Ltd., Beijing, China). Following the manufacturer’s instructions, the cDNA was created by reverse transcribed cellular RNA samples for 30 min at 42 °C using the cDNA Reverse Transcription Kit (Vazyme Biotech Co., Ltd., Nanjing, China). PCR amplification was carried out in a volume of 20 μL using AceQ qPCR SYBR Green Master (Vazyme Biotech Co., Ltd., Nanjing, China), and β-actin was chosen as the reference gene. The following protocol was used to perform quantitative PCR amplification on each sample using a fluorescence quantitative PCR instrument (QuantStudioTM 6 flex, ABI, Los Angeles, CA, USA): 40 cycles each of 95 °C for 35 s, 60 °C for 30 s, and 72 °C for 15 s [20]. Gene-specific PCR primers were synthesized commercially (TsingKe Biological Technology, Wuhan, China) and shown in Table S1.

### 2.9. Molecular Docking

The structures of PAA and PAB were constructed using SYBYL 2.0, and the optimized stable conformations were saved in mol2 format. The crystal structures of cancer-related proteins (Figure S1) and their ligand structures were downloaded from the PDB database and imported into SYBYL. The docking module in the application was used to modify the structure of all proteins, remove water molecules and other ligands, and hydrogenate. The docking active pockets based on the original ligand structures were created. The processed proteins were stored and prepared for subsequent molecular docking. The Surflex-Dock program in the SYBYL 2.0 software was used to perform flexible docking between PAA, PAB and 15 processed proteins. The affinity between the small molecule ligand and the receptor protein was analyzed using the Total Score function in the docking module. Docking of triterpene proteins was completed by using AutoDock Vina software. The minimum binding energy was used as the evaluation index, and the one with the smallest binding energy was selected for visualization and analysis using Pymol 2.6 software and LigPlot 1.4.5 software [21].

### 2.10. Statistics Analysis

Data from the experiments were represented as mean values ± SD. The Tukey’s test for comparisons was used in the statistical analysis using SPSS 19. One-way analysis of variance (ANOVA) was used to calculate significant differences at *p* < 0.05.

## 3. Results and Discussion

### 3.1. Antiproliferative Activity of PAA and PAB on Human Cancer Cells

**Figure 2:**
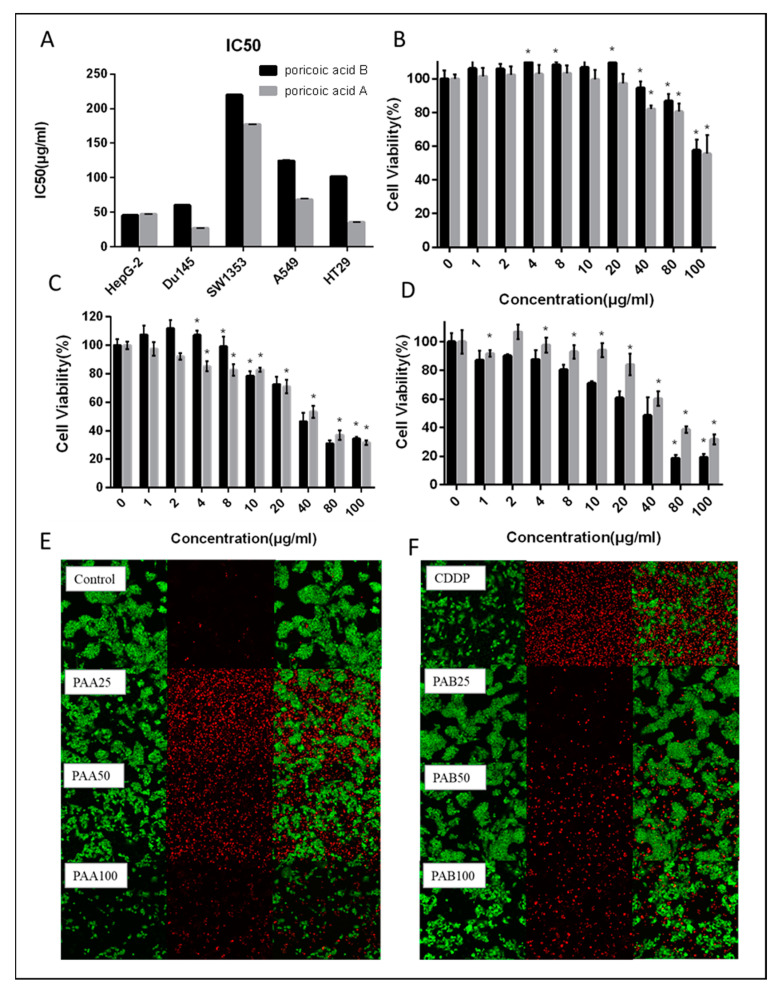
(**A**) IC50 values of PAA and PAB on tumor cell lines of different systems for 48 h; the effects of different concentrations of PAA and PAB on HepG2 cell viability for 24 h (**B**), 48 h (**C**) and 72 h (**D**); (**E**,**F**) the apoptotic effects of PAA and PAB at different concentrations on HepG2 cells were observed by fluorescence microscopy with AO/EB staining. * with column means significant difference compared with the control group (Duncan’s multiple range test at *p* < 0.05).

The MTT assay was used to determine the inhibitory activity of PAA and PAB on five human cancer cell lines from different systems [22]. The results are shown in Figure 2A. PAA and PAB had inhibitory effects on HepG2, A549, HT29, DU145, SW1353 cells, but with a significant difference. PAA and PAB had more obvious inhibitory effects on HepG2 and DU145, and the half inhibition rate of osteosarcoma cells was higher than that of other system cells. Compared with PAB, PAA had higher activity in inhibiting tumor cells, especially the most significant effect on DU145. Therefore, HepG2 cells were used in subsequent experiments. The time–effect relationship between PAA and PAB was further investigated (Figure 2B–D). There were dose-dependent and time-dependent declines in cell viability. The viability of tumor cells decreased significantly with the prolongation of administration time or the increase in drug concentration (*p* < 0.05). The cell viability had an obvious time-dose-effect relationship in the range of 4–80 μg/mL. In addition, the anti-tumor effects of three other triterpene compounds obtained from *Poria cocos* were also studied. After administration of 100 μg/mL *Poria cocos* triterpene monomer for 24, 48 and 72 h, the viability of tumor cells was above 80% with no significant anti-tumor effects.

The inhibitory effects of lanostane triterpenes on tumor cells are dependent on structures. The structure of lanostane triterpenoids in *Poria cocos* bark includes ring-opening lanostane triterpenoids and closed-ring lanostane triterpenoids [23]. Most lanostane triterpenes have been reported to have inhibitory activities against tumor cells *in vitro* [5]. For the same type of lanostane triterpene, the substituents also have a significant effect on its activity [6]. Studies have shown that the ring-opining lanostane triterpenes are less cytotoxic than the closed-ring structure. In terms of the five triterpenes obtained in the experiment, the open-loop structure had a more significant inhibitory effect on cells than the closed-loop structure. The hydroxyl group at the C16 position can increase the toxicity of triterpenes to tumor cells [24]. Studies have also shown that double bonds in the B and C rings could increase cytotoxicity [6]. Compared with the structures of PAA, PAB and other triterpene monomers, the double bond at position 24 was also beneficial for tumor inhibition.

In order to verify the cell apoptosis process, AO/EB staining was used to further observe the changes in cell morphology. Acridine Orange can freely penetrate the intact cell membrane, combine with DNA in the nucleus, and excite green fluorescence. Ethidium bromide cannot penetrate cells with intact membranes. It can only enter cells when the cell membrane is damaged and the permeability increases, interacting with nuclear DNA and producing orange-red fluorescence [25]. Cisplatin, the most commonly used anti-tumor drug in chemotherapy for various malignant tumors, was used as a positive control [26]. As the results in Figure 2E,F show, cells in the control group showed different shades of green fluorescence. The red fluorescence increased and the green fluorescence dropped after 25 g/mL PAA and PAB treatment, indicating that the membrane permeability increased and the cells significantly displayed apoptotic features (*p* < 0.05). The effect of PAA was more obvious than the same dose of PAB, and apoptotic cells with orange chromatin could be observed.

### 3.2. Effect of PAA and PAB on Cell Cycle Arrest and Apoptosis

**Figure 3:**
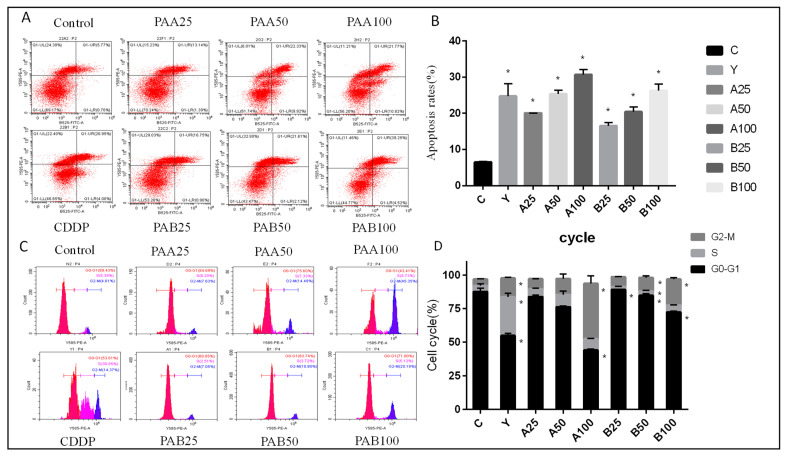
The effects of different concentrations of PAA and PAB on HepG2 cell apoptosis (**A**,**B**) and cycle (**C**,**D**). * with column means significant difference compared with the control group (Duncan’s multiple range test at *p* < 0.05).

Apoptosis, also known as programmed cell death, significantly affects the balance between cell survival and death [27]. It has been extensively investigated how triterpenes cause cell apoptosis [22]. To identify apoptosis in living cells, annexin V-FITC/PI labeling was utilized [28]. As can be seen from Figure 3A,B, the majority of the control group’s cells were normal. The number of early apoptotic and late apoptotic cells increased with the increase in drug concentration. The apoptosis rate of PAA-treated cells was 14.53% when the drug concentration was 25 μg/mL. The maximum apoptosis rate was 32.59% when treated with 50 μg/mL drug. After PAB was incubated with tumor cells, the dose–response relationship was obvious (*p* < 0.05). The rate of early apoptotic cells was 21.61%, and 2.12% of cells were late apoptotic when treated with 50 μg/mL of the drug, which was 17.20% higher than the total apoptotic cells in the control group.

According to the results, the dose-effect relationship of PAB was demonstrated. However, the concentration of PAA above 50 μg/mL showed no dose–response relationship. At low dosages, PAA increased the amount of apoptosis, whereas PAB had a stronger effect at high doses, which was superior to the positive control medications. In addition, PAA induced late apoptosis significantly more than PAB (*p* < 0.05). According to the findings, the anti-tumor mechanism of triterpenoids in *Poriae cutis* might be through inducing apoptosis of tumor cells [1].

We used PI staining to assess the effect of terpenes on the cell cycle. As drug concentrations rose, apoptotic cell peak was increased, indicating that apoptosis was enhanced. As can be seen from the Figure 3C,D, when the drug concentration was 25 μg/mL, there was a 3.00% increase in G2/M phase cells compared to the control group. The number of G2/M phase cells rose by 9.88% in comparison to the control group when the concentration of PAA reached 50 g/mL. After 100 μg/mL drug incubation, the number of cells in the G2/M phase increased by 11 times more than the control group. The dose–effect relationship of G2/M phase arrest was obvious after PAB administration (*p* < 0.05). After high-dose administration, the number of cells in the G2/M phase was five times higher than that of the control group.

After medicines were added, the cell cycle was disrupted, and the proportion of cells in the G2/M phase rose. It reduced HepG2 cell synthesis and protein synthesis, which in turn slowed down cell proliferation, thus exerting an anti-tumor effect. Xu et al. (2019) found that triterpenoids from natural products also halted the cell cycle in the G2/M phase [28]. Other studies have shown that triterpenoids can increase the number of G1 phase cells in tumors [29]. The various structural and functional groupings of triterpenoids may be connected to this.

### 3.3. Effect of PAA and PAB on ROS Production in HepG2 Cells

The intracellular esterases and reactive oxygen species can hydrolyze the fluorescent probe DCFH-DA to produce non-fluorescent DCFH, which can then be oxidized to fluorescent DCF by the intracellular esterases [30]. The higher the concentration of reactive oxygen species, the stronger the fluorescence intensity of DCF. It was found that almost all tumor cells have a higher metabolic rate compared to normal cells, resulting in high concentrations of ROS production by the tumor cells themselves [31]. Therefore, tumor cells have a higher sensitivity to ROS stimulation.

**Figure 4:**
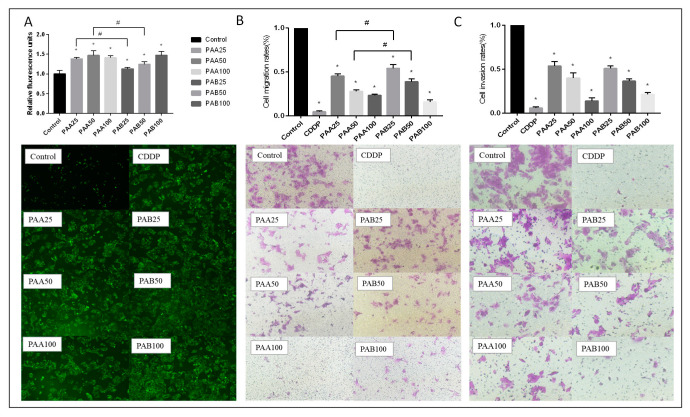
The effects of different concentrations of PAA and PAB on HepG2 cell ROS production (**A**), migration (**B**) and invasion (**C**). * with column means significant difference compared with the control group, # means significant difference between the two groups connected by the line (Duncan’s multiple range test at *p* < 0.05).

Therefore, the production of reactive oxygen species after triterpene stimulation of cells was measured. The results in Figure 4A showed that the ROS levels of cells increased significantly (*p* < 0.05) after PAA and PAB were administered in a dose-dependent manner. Fluorescence microscopy was used to observe the reactive oxygen species generation. The results also showed that PAA and PAB stimulated the production of reactive oxygen species in hepatocellular carcinoma cells, as shown by the increasing green fluorescence. Therefore, it is possible to selectively kill tumor cells by increasing ROS in tumor cells with low harm to normal cells [32,33].

In our previous studies, it was shown that triterpenes promote the production of TNF-alpha, which plays a role in cell death and tumor progression [12]. It has been shown that tumor necrosis factor alpha can act directly on tumor cells and induce ROS within them, which is the rationale behind the use of cellular immunotherapy for targeted elimination of cancer cells [34]. ROS are considered to be tumor-suppressing agents, as several chemotherapeutic agents have been reported to promote ROS production and activate cell death [35]. The main physiological role of ROS is their ability to regulate multiple signaling pathways acting on tumor cells, including NF-κB, MAPK, p53, Keap1-Nrf2 and PI3K/AKT.

### 3.4. Effect of PAA and PAB on Migration and Invasion in HepG2 Cells

Cancer is difficult to cure. Due to the uncontrolled expansion of cancer cells, which makes it difficult to prevent the death of immune cells [36], cancer cells may spread concurrently to other areas of the body where they might colonize, develop, and form metastases [37]. Thus, in the course of cancer treatment, it is crucial to restrict the metastasis and invasion of cancer cells. This study demonstrated that triterpenes from *Poriae cutis* can effectively induce apoptosis in hepatocellular carcinoma cells, but it was not clear whether they had the property of inhibiting metastasis. Since HepG2 cells are typical epithelial cancer cells with invasive metastatic properties, they have been widely used in cancer cell invasion and metastasis studies [38]. In this study, HepG2 cells were used as a model to investigate the effect of triterpenes from *Poriae cutis* on invasion and metastatic ability.

Figure 4B showed the results of cell migration, where the top figure showed the quantitative analysis of crystalline violet staining and the bottom figure showed the qualitative analysis. According to the results, cell migration was inhibited by PAA and PAB in a concentration-dependent manner (*p* < 0.05). Figure 4C showed the results of cell invasion. Similar to migration, the ability of PAA- and PAB-treated cancer cells to invade the membrane was also inhibited depending on the concentration (*p* < 0.05). The simulated membrane could be penetrated by cancer cells in the control group, and both the number of migrating cells and the force of invasion were higher. The invasion force was decreased after the triterpene intervention.

### 3.5. Mechanisms of Apoptosis Induced by PAA and PAB in HepG2 Cells

In order to further determine the mechanism of the anti-tumor effect of PAA and PAB intervention, qRT-PCR was used to evaluate the expression of genes related to apoptosis, cell cycle, invasion, and migration in HepG2 cells.

**Figure 5:**
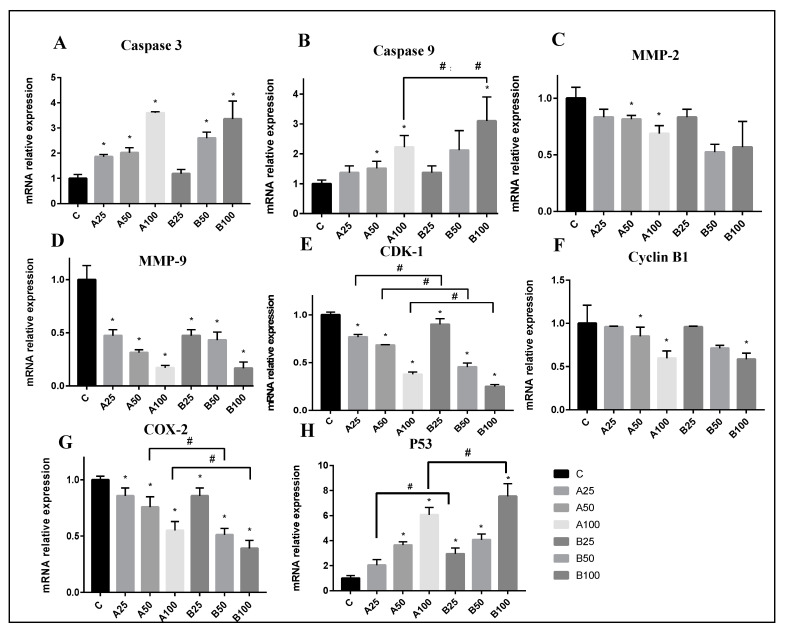
The effects of PAA and PAB on HepG2 cells for 48 h on the expression levels of related mRNAs (**A**–**H**) were analyzed by qrt-PCR. * with column means significant difference compared with the control group, # means significant difference between the two groups connected by the line (Duncan’s multiple range test at *p* < 0.05).

The caspase family is a very important protein related to apoptosis, and various apoptotic pathways eventually activate the caspase family [39,40]. The expression of caspase-3 and caspase-8, the key regulatory proteins of apoptosis in tumor cells, were analyzed. Results were shown in Figure 5A,B. Compared with the control group, the mRNA expressions of caspase-3 and caspase-8 in the drug intervention group were significantly increased (*p* < 0.05), with a dose-response relationship. The protein expression level of caspase-3 increased slightly after the effect of PAA, which was more obvious than that of PAB. One of the caspase cascade’s most downstream elements is caspase-3 [41]. Caspase-8 is a regulatory protein of the apoptotic exogenous pathway and caspase-9 is a regulatory protein of the apoptotic endogenous pathway [42,43]. The activation can further initiate caspase-3. The increase in the expression of caspase-8 indicated that it was mainly involved in the apoptosis process of HepG2 cells through exogenous pathways. Caspase-3 is a crucial, essential executive molecule that is involved in DNA fragmentation, chromatin condensation, and the creation of apoptotic bodies [44]. After being activated by triterpenoids from *Poriae cutis*, caspase-3 can participate in the cleavage of the corresponding cytoplasmic nucleus, thereby causing cancer cells to undergo apoptosis.

For cancer cells to migrate and spread, epithelial calcium adhesin (E-cadherin) is the main factor. Studies have shown that E-cadherin expression deficiency is prevalent in cancerous cells [45]. After the intervention of triterpenes from *Poriae cutis*, E-cadherin protein expression dramatically increased. By increasing the expression level of E-cadherin, the migration and motility of cancer cells were inhibited. After the cells were released from the adhesion, the invasion of the extracellular matrix required the degradation of extracellular matrix components by hydrolases, such as the matrix metalloproteinase family (MMPs) [46]. MMPs’ proteins, particularly MMP-2 and MMP-9, have been found to be crucial for tumor invasion and migration [47,48]. After the triterpenoid intervention, MMP-2 and MMP-9 mRNA expression reduced. The PAA and PAB groups’ expression of MMP-9 reduced more sharply than that of the control group (*p* < 0.05), with a dose-effect relationship (Figure 5C,D). According to the study, liver cancer cells have abnormally high levels of MMP-9 rather than MMP-2 when compared to normal liver cells [49,50]. Further research involved measuring MMP-9 protein expression. The outcomes showed that the PAA group inhibited protein expression more obviously than the PAB group.

Cell cycle regulation primarily consists of two components: cell cycle proteins and cyclin-dependent kinase (CDK), as is well known [51]. After the injury, the members of the upstream pathway are activated or inactivated successively and, finally, the process of the cell cycle is determined by the sensing signal of the CDK-cyclin complex [51]. The CDK1 complex, also known as a mitosis-promoting factor, is a key regulator of the G2/M phase [52]. The expression of the cyclin B1 gene in tumor cells was downregulated by medium to high doses of PAA and PAB (*p* < 0.05). Moreover, the expression of CDK1 mRNA in the cells was significantly decreased (Figure 5E,F) and there was a significant difference between equal concentrations of PAA and PAB groups (*p* < 0.05). Downregulating the expression of this gene can inhibit cell proliferation and cause G2/M phase arrest. This explains why the cells in the above study were halted in G2/M phase.

Cyclooxygenase-2 (COX-2) and COX-1 are two isoenzymes found in mammals [53]. COX-1 is a structural enzyme that maintains the normal physiological functions of the organism [54]. COX-2 is an inducible enzyme that can be stimulated by growth factors, inflammatory mediators, and cancer-promoting agents to be highly expressed [55,56]. In normal cells, COX-2 generally maintains a low level or does not express, and oxidative stress and other stimuli increase the level of the COX-2 protein [55]. The highly expressed COX-2 protein exists in tumor tissues or cells [57]. COX-2 can promote tumor cell invasion and metastasis, resist apoptosis, and improves tumor drug resistance [58,59]. The experimental results showed in Figure 5G that the mRNA expression of COX-2 in tumor cells decreased with the increase in drug administration concentration, and the effect was more significant in medium and high concentrations (*p* < 0.05). Inhibition of COX-2 protein expression is likely to inhibit carcinogenesis and attenuate tumor growth [60].

P53 is a very important tumor suppressor. Mutations in this gene occur in more than 50% of all malignant tumors [61]. The p53-mediated cell signaling pathway plays an important role in the regulation of normal cellular life activities [62,63]. When cells are stimulated by DNA damage, growth factor deficiency or hypoxia, the p53-mediated signaling pathway can regulate cell cycle progression and apoptosis by regulating its downstream factors [64]. Following the action of PAA and PAB, both P53 mRNA expression were significantly activated in cancer cells as shown in Figure 5H (*p* < 0.05), with a dose-effect relationship for its mRNA expression. Studies have shown that p53 can cause G2/M cycle arrest by regulating Cyclin B1, GADD45, and other downstream factors [63].

### 3.6. Screening by Molecular Docking Simulation

A total of fifteen protein targets related to AKT/PI3K and MAPK (mitogen-activated protein kinases) pathways as well as those related to apoptosis, cell cycle, invasion and metastasis were selected for molecular docking. The Total Score function takes into account hydrophobicity, polarity, entropy, and solubilization, and the larger the value, the higher the affinity between the ligand and the protein. In addition, the more the Crash value tends to zero, the less likely the Total Score value is to be a false positive. The Polar value is used to exclude the docking results without hydrogen bond formation between the ligand and the protein. The molecular docking results are shown in Table 1. JNK, ERK and P38 proteins in the MAPK pathway, as well as AKT and PI3K proteins, have strong affinity with PAA and PAB. The highest Total Score value of 7.1757 was obtained for the interaction of PAB with the P53 protein. However, the Total Score values between PAA with Bax and Bcl-2 were lower, both less than four. Furthermore, the affinity between PAA and PAB with cell migration and invasion-related proteins was relatively weak. Molecular docking results also showed that PAB had a more pronounced effect on cycle-related proteins compared to PAA. AutoDock was used for molecular docking to evaluate the binding degree of ligand and receptor molecules in terms of their binding free energy. The smaller the binding free energy, the better the binding effect and the stronger the interaction between the two [65]. The free energy of binding for each protein to PAA and PAB was less than −5.0 kcal/mol, as shown in Table 1, with JNK and AKT having the highest affinity.

The signaling pathways PI3K/Akt and MAPK are crucial in the regulation of cancer and development [66,67]. The PI3K/AKT pathway regulates cell survival and apoptosis by regulating the activation state of multiple downstream effectors [68]. Activation of PI3K/AKT promotes cell survival and proliferation and resists apoptosis [69]. It has been shown that various triterpenoids act through AKT/PI3K to influence the proliferation and cycle of tumor cells [70]. For example, uvaol produces a cellular block and boosts apoptosis in cancer cells by upregulating the PI3K/AKT signaling pathway and downregulating the expression of the anti-apoptotic protein Bcl2 and pro-apoptotic protein Bax, respectively [70]. The effects of PAA and PAB on the expression of caspase family histones and P53 may be affected by the downregulation of the upstream AKT/PI3K signaling pathway. In most cancer cells, the Akt signaling pathway also plays an important role in the G2/M phase and in apoptosis and survival [71]. Chen et al. (2015) found that flavin compounds can induce G2/M phase arrest in cells by regulating the Akt pathway [72]. In addition, inhibition of Akt activation can also inhibit cancer cell migration by upregulating E-calmodulin, β-linked protein and downregulating the mesenchymal cell waveform protein [73,74]. Therefore, the downregulation of AKT/PI3K protein expression and the expression of downstream caspase, CDK-Cyclin protein, MMP and other proteins, ultimately promote apoptosis.

A set of serine-threonine protein kinases known as MAPK can be activated by a variety of extracellular stimuli, including cytokines, neurotransmitters, hormones, cellular stress, and cell adhesion [75]. They participate in the control of a number of biological processes, including cell division, proliferation, and apoptosis [76,77]. The ERK, JNK, p38 MAPK, and ERK5 pathways make up the majority of the MAPK pathway [76,78]. Growth factors or mitogens are the main sources of ERK 1/2 activation, which promotes cell differentiation [79], growth and survival, whereas oxidative stress and cytokines preferentially activate JNK and p38, causing inflammation and apoptosis [80]. Apoptosis can be induced by the JNK and p38 pathways, while being inhibited by the ERK pathway [80].

There are few reports on the interaction of AKT/PI3K and MAPK pathways in suppressing cancer development. Studies have shown that inhibition of Erk1/2 and JNK hindered Akt activation to a certain extent, while inhibition of p38 had no significant effect on Akt [81]. In contrast, inhibition of PI3K/Akt significantly inhibited JNK activation, while other proteins had no effect [69]. This suggests that PI3K/Akt and MAPK pathways interact with each other to mediate apoptosis. However, whether there is a reciprocal or parallel relationship between them and the role of triterpenoids in the induction of hepatocellular carcinoma cell apoptosis still needs to be further explored.

Moreover, the triterpenoids consist of six isoprenoid (2-methyl-1,3-butadiene) ketone units, whose highly saturated carbon skeleton provides a large number of protons for the production of deuterium-depleted metabolic water by mitochondria. Natural triterpenoids can be used as ketogenic substrates by cells with lower deuterium content than cytoplasmic water, thus aiding mitochondrial NADPH-dependent macromolecular synthesis, including DNA [82]. In fact, it has been demonstrated that deuterium-depleted water inhibits lung tumor growth *in vivo* by lowering proliferation in the A549 cell line, while enhancing apoptosis [83]. Deutenomics research has also been used to study colorectal cancer. Essential ketogenic branched-chain amino acids are directly converted to succinyl-CoA and acetyl-CoA in mitochondria, where deuterium-depleted proton transfer from nutrients to metabolic substrate water is provided by natural ketogenic substrates. Depletion of deuterium isoforms in nucleotides prevents transformation of colon cells due to cycle arrest [84]. Deuterium depletion caused by the oxidation of natural cellular ketogenic substrates can prevent or reverse the development of breast cancer and is of increasing importance [85]. Studies of biological deuterium fractionation and identification techniques have indicated that deuterium-depleted fatty acids enter cells through the circulation and act as an intermediate proton carrying carbon source for mitochondrial respiration [86]. Extracellular deuterium depletion may serve as a metabolic therapeutic adjuvant that can be initiated by a diet with depleted water drinking [86,87]. In these subsequent investigations, triterpenes were further examined *in vivo* for their affect on cancer metabolism-related multiple metabolites and to evaluate the deuterium depletion potential of PAA and PAB during metabolic water formation.

## 4. Conclusions

In this study, two triterpenoids, PAA and PAB, were isolated from *Poriae cutis* by high-speed counter-current chromatography, and their anti-tumor activities were studied. Both PAA and PAB showed significant inhibitory effects on a variety of cancer cells *in vitro*. PAA and PAB mainly inhibit the activities of cancer cells by inducing apoptosis, inhibiting migration and invasion, regulating G2/M phase arrest, and stimulating elevated ROS levels. Through experiments and computer simulations, it was found that PAA and PAB have strong affinity for AKT/PI3K and JNK pathways. It can be speculated that due to their structural differences, PAA and PAB may regulate downstream pathway proteins by regulating the AKT/PI3K and MAPK pathways, but with different affinities for each target. In conclusion, the triterpenoids PAA and PAB from *Poriae cutis* have great potential in inhibiting the occurrence and development of tumors.

## Figures and Tables

**Table 1:** Docking results of PAA and PAB with cancer-related proteins.

Proteins	Poricoic Acid A (PAA)				Poricoic Acid B (PAB)			
	Total Score	Crash	Polar	Binding Energy (kcal/mol)	Total Score	Crash	Polar	Binding Energy (kcal/mol)
AKT	4.4734	−1.6060	5.5532	−7.6	4.3085	−1.4271	4.8327	−8.0
PI3K	4.2297	−2.8787	4.7772	−5.1	4.1559	−1.4352	3.7563	−6.7
ERK	4.4894	−3.2933	3.7209	−7.4	4.0913	−1.6300	2.6628	−6.5
JNK	4.1819	−1.8209	2.3171	−8.8	4.8064	−1.3607	1.9971	−8.2
P38	5.6784	−2.1307	4.0457	−8.0	5.9058	−2.2057	3.3674	−6.9
P53	4.5928	−3.1685	3.1603	−6.5	7.1757	−2.8259	4.3213	−7.2
Bcl-2	2.3230	−1.5790	2.7255	−6.9	3.2755	−0.8705	3.9976	−7.5
Bax	1.4245	−1.5416	1.7694	−6.7	4.4059	−0.8569	4.6240	−6.2
Caspase-3	5.1799	−2.2216	5.6010	−6.9	4.5097	−1.6615	5.0136	−7.1
E-cadherin	3.0189	−2.0251	3.9197	−6.4	3.3511	−0.9367	4.9135	−6.2
ICAM-1	3.0536	−2.7535	4.3288	−6.1	3.1307	−0.9398	2.4113	−5.9
MMP-9	5.4450	−3.4685	3.3047	−7.6	6.4213	−1.8768	2.5850	−6.5
COX-2	3.7997	−2.4828	1.5334	−7.1	4.9986	−2.0211	2.3840	−8.3
Cyclin D	4.6249	−1.8272	2.9440	−6.5	5.5773	−1.3391	2.8638	−6.5
CDK1	3.9919	−1.9891	3.9244	−7.8	5.5295	−1.8477	4.5974	−7.9

## Supplementary Materials

The following supporting information can be downloaded at: https://www.mdpi.com/article/10.3390/nu15204360/s1, Table S1. PCR primer sequences. Figure S1. the crystal structures of cancer-related proteins.
