# Supplementary material for: Regulation of Tumor Apoptosis of Poriae cutis-Derived Lanostane Triterpenes by AKT/PI3K and MAPK Signaling Pathways In Vitro
Source: Nutrients. 2023 Oct 13;15(20):4360. doi: 10.3390/nu15204360 (PMC10610537; doi:10.3390/nu15204360)
Supplement: Supplementary file 1 [file nutrients-15-04360-s001.zip › nutrients-2640781-supplementary.pdf]

Table S1 PCR primer sequences.

Gene	Fonvard primer (5' to 3')	Reverse primer (5' to 3')
β-actin	CGTGCTCAGGGCTTCTTGT	CACCTGGGTCATCTTCTCG
Caspase 3	TTTGTTTGTGTGCTTCTGAGCC	GATGTTCTGGAGAGCCCCG
Caspase 8	GATGTTCTGGAGAGCCCCG	TTGACGTCTGTGGTCCGTCC
MMP-2	ATGACAGCTGCACCACTGAG	ATTTGTTGCCCAGGAAAGTG
MMP-9	TTGACAGCGACAAGAAGTGG	GCCATTCACGTCGTCCTTAT
CDK4	TTTTGAGACCAGGGCTTGC	AGGTGCGTGCCTGTAGTGT
COX-2	TTCATGATCACGCCCTCATA	TAAAGGATGCGTAGGGATGG
cyclin E	CCTGGATGTTGACTGCCTTGA	CGCACCACTGATACCCTGAAA
cyclin D1	GCGAGGAACAGAAGTGCG	GGAGTTGTCGGTGTAGATG

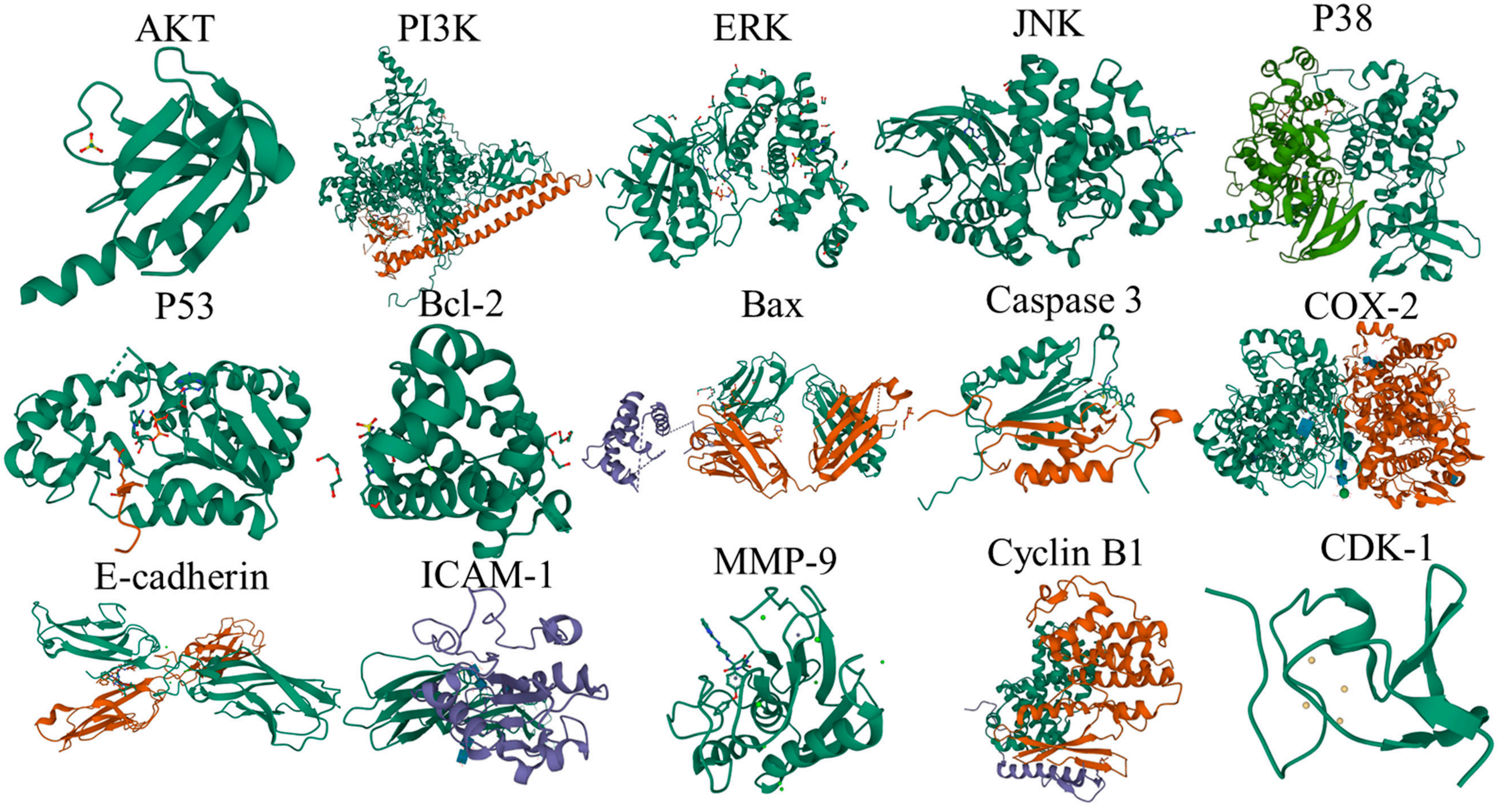

Figure S1 the crystal structures of cancer-related proteins
